# Impact of Extremes of Body Mass Index (BMI) in End-Stage Renal Disease (ESRD) Patients

**DOI:** 10.7759/cureus.25892

**Published:** 2022-06-13

**Authors:** Rizwan Rabbani, Edva Noel, Suzanne Boyle, Waqas Ahmad Khan, Paul Pronovost, Avrum Gillespie

**Affiliations:** 1 Nephrology, Temple University Hospital, Philadelphia, USA

**Keywords:** obesity paradox, obese, bmi, hemodialysis, esrd, prognosis

## Abstract

The principal objective of this systematic review is to determine the prognosis of end-stage renal disease (ESRD) patients on maintenance hemodialysis with high body mass index (BMI) and study the potential mechanisms behind it. PubMed and Google Scholar electronic databases covering the period of the last 30 years 1992 to 2022 are searched thoroughly and a total of 11 articles were finally selected for the study. Reference lists of included papers are also searched. Each paper was examined by two independent evaluators who also extracted data from full papers. The quality of the selected studies was assessed by different quality assessment tools and only moderate- to high-quality papers are included.

In this systematic review, we studied different mechanisms explaining the obesity paradox in patients on maintenance hemodialysis, i.e., hemodynamic stability, the concentration of TNF-α receptors, neurohumoral response, role of inflammation, blood pressure, etc. also, the effect of age, gender, duration of treatment, acetyl-ghrelin on obesity paradox have been considered in our paper. This systematic review demonstrates the evidence of an inverse relationship between BMI and all-cause mortality in ESRD patients on maintenance hemodialysis.

## Introduction and background

Body mass index (BMI) is basically a measure of body fat based on weight and height. According to the World Health Organization (WHO), a BMI above 25 is considered to be overweight, and above 30 is obese, whereas a BMI below 18.5 is considered underweight [1]. In chronic disease populations such as chronic obstructive pulmonary disease, heart failure, chronic kidney disease (CKD) patients on maintenance hemodialysis, and peripheral vascular disease, the deleterious effect of low BMI on survival has been recognized [2]. People with high BMI are at high risk for developing non-communicable diseases such as cardiovascular diseases, primarily hypertension, heart disease, and stroke; diabetes; musculoskeletal disorders, mainly osteoarthritis; as well as some cancers. [3] Recent evidence suggests that high BMI is also one of the strongest risk factors for new-onset CKD. This association between obesity and CKD is thought to be due to compensatory hyperfiltration and increased intraglomerular pressure, ultimately causing kidney damage [4].

However, contrary to the general population, obesity is linked to better survival in hemodialysis patients. This phenomenon is called the “obesity-survival paradox” [4]. There are several possible explanations for this phenomenon, such as stable hemodynamic status and high levels of circulating TNF-α receptors in obesity, diminished neuro-hormonal response in obese individuals, etc. In this systematic review, we have explained these possible mechanisms in detail.

## Review

Methodology

Study Protocol

As a part of this study, the PRISMA (preferred reporting items for systematic and meta-analyses) guidelines was used.

Source of Data Collection

To write this article, PubMed and Google Scholar were used as databases. Data published over the last 30 years, i.e., 1992 to 2022, were reviewed in a multidimensional and systematic manner to recognize relevant articles. As a part of the data collection, in PubMed, MeSH (medical subject headings) keywords and regular keywords were used. For Google Scholar, regular keywords were used.

Inclusion and Exclusion Criteria

Four inclusion criteria have been implemented to collect the data: i) to include studies conducted in the past 30 years with an emphasis on newer studies, ii) to integrate studies that were carried out only on the patients who were on hemodialysis, iii) to include the studies conducted exclusively on humans, and iv) to select the studies written in English.

As for the exclusion criteria, studies conducted on patients who were on peritoneal dialysis were excluded.

Search Content

To identify the articles, the following keywords have been used. 

MeSH keywords: (("Kidney Failure, Chronic"[Majr]) AND "Body Mass Index"[Mesh]) AND "Renal Dialysis"[Majr]

All the articles have been reviewed and only the appropriate articles have been chosen.

Ethical Issue

This study aims to review all of the published data systematically. To achieve this, all the articles were collected in an accurate, adequate, and systematic manner.

Quality Check

The quality of the included articles was assessed using the Newcastle Ottawa scale for cohort and case-control studies. NIH quality assessment tool was used for a cross-sectional study. Also, the quality of systematic/meta-analysis reviews was assessed by AMSTAR-2. All the low-quality articles have been omitted and only moderate and high-quality papers were incorporated.

Data Search

**Figure 1:**
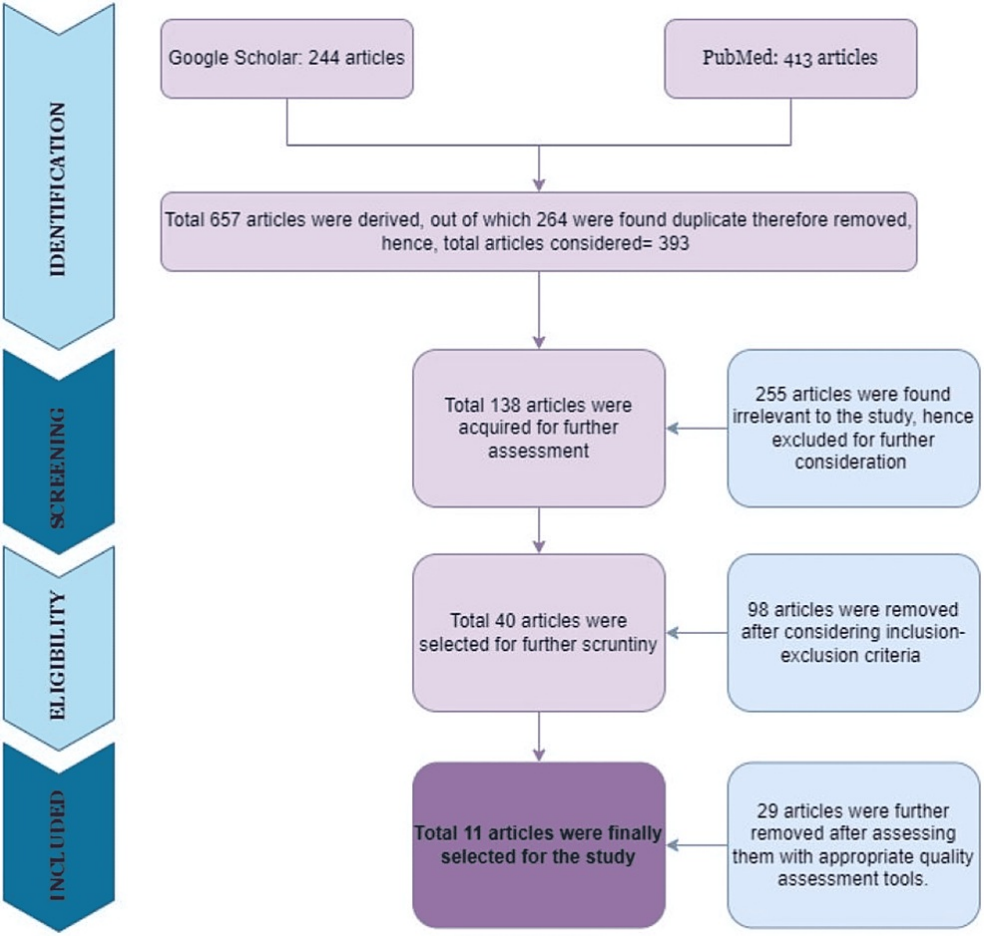
Prisma Flow Diagram

With the help of MeSH keywords and regular keywords, a total of 657 articles were found. The majority of the articles, 413 to be precise were from PubMed, and the remaining 244 were from Google Scholar. Two hundred and sixty-four duplicate articles were eliminated. An additional 255 articles were removed as they were found not to be relevant to our study. A total of 138 full-text articles were assessed for further consideration. Ninety-eight of these articles were excluded after considering the inclusion-exclusion criteria, and 40 articles were obtained for further analysis. After using suitable quality assessment tools, another 29 articles were excluded. And finally, 11 articles were gathered up for the study. This entire process to recognize relevant articles for our study has been represented in Figure 1.

Discussion

Individuals with higher BMI up to 45kg/m^2^ have demonstrated better survival and lower cardiovascular mortality. Whereas low body weight is strongly related to an increased risk of cardiovascular as well as all-cause mortality. Several possible explanations for this obesity paradox have been suggested [4].

Hemodynamic Stability

A large number of patients on hemodialysis suffer from some degree of heart failure or they are in a relative state of fluid overload [5]. A study conducted by Oreopoulos suggested that when compared with chronic heart failure patients with normal BMI, obesity (RR 0.60, 95% CI 0.53-0.69) and overweight (RR 0.81; 95% CI 0.72-0.92) are linked with lower cardiovascular (cv) mortality, whereas underweight or low-normal-weight is associated with relatively higher cv mortality (RR 1.20, 95% CI 1.04-1.38) [6]. Although obese and overweight patients with heart failure have similar pulmonary capillary wedge pressure (PCWP) and cardiac indexes, they tend to well maintain hemodynamic stability [5]. 

*Role of Tumor Necrosis Factor (TNF)-α Receptors* 

Heart failure patients and patients on hemodialysis tend to have elevated levels of circulatory TNF-α, particularly, in those who have intermittent episodes of fluid overload. This TNF-α can cause cardiac injury by its negative ionotropic and proapoptotic effect. The adipose tissue generates soluble TNF-α type I and type II receptors, causing higher circulating levels of both the receptors in overweight and obese patients. These receptors neutralize the undesirable biological effects of tissue necrosis factor-α, hence TNF-α receptors play a cardio-protective role. This alteration in TNF-α system may confer survival benefits to obese patients [7].

Altered Neurohormonal Response

Obesity is linked with altered sympathetic and renin-angiotensin systems. A comparison study carried out on lean and obese subjects showed that lean subjects have markedly elevated levels of plasma adrenaline and renin during exercise, despite a similar baseline value. Elevated sympathetic and RAS activities are linked with poor outcomes in heart failure and the fluid overload state seen in hemodialysis patients. Hence, decreased responsiveness of these neuro-hormonal systems may confer a favorable prognosis in obese patients on hemodialysis. However, some observational studies have demonstrated that obese patients on hemodialysis have relatively lower blood pressure readings when compared with underweight patients on hemodialysis [8].

Protective Role in Inflamed Patients

Inflammation in hemodialysis patients can be due to processes associated with kidney failure itself, or it can be a result of dialysis, or it can be due to other reasons such as oxidative damage, impaired immune function associated with uremia, oxidative damage, protein-energy malnutrition, dialysate, a build-up of glycation end products, back leak of dialysate, vascular access, and old age. High BMI plays a protective role in inflamed dialysis patients. There are several mechanisms that explain this theory. While high BMI may be linked with catabolism and muscle wasting, it probably reflects the preserved energy reserves and the preserved appetite. When kidney function declines and uremia develops, these preserved energy reserves become very essential. Secondly, as the production of uremic toxin is relatively higher in lean/low body weight patients, inflamed patients with high BMI have a better outcome. Lastly, since the density of endothelial progenitor cells is linked to obesity, there is a possibility that the endogenous repair mechanisms are well preserved in patients with high BMI [9]. 

Malnutrition-Inflammation Complex (MIC) Syndrome

Unintentional weight loss has always been a marker of poor health. Decreased food intake can be seen as a result of anorexia due to uremia, high pill burden, and volume overload. Elevated catabolism is due to frequent infections, oxidative stress, and cachectic inflammatory states [5]. Hence, maintenance of adiposity or weight gain will reflect the absence of these risk factors and better overall health. In patients with hemodialysis, inflammation and malnutrition are very closely linked. This MIC syndrome might be associated with cardiovascular and all-cause mortality [10,11].

Blood Pressure in Obesity

Evidence suggests that HD patients with high blood pressure have a better prognosis than patients with normal blood pressure. It is possible that the inverse relationship between obesity and mortality may be related to hypertension. The better control of hypertension among obese patients may be due to the following reasons.

First of all, obese patients can sequester excessive fluid volume in extracellular space more efficiently than lean individuals, and as a result, not get hypertensive. Secondly, an increase in muscle mass might be associated with an increased expression of release in patients with high BMI. Renalase is a catecholamine metabolizing enzyme that is predominantly expressed in the skeletal muscles and can decrease the circulating catecholamine levels. This might be associated with a lower prevalence of hypertension and better control in obese subjects [12].

Reverse Causation

There is a possibility that BMI may not be a cause but a consequence of the conditions that result in poor outcomes in hemodialysis patients. Comorbid conditions can lead to wasting such as cardiac cachexia and also a high mortality rate. However, even though the reverse causation is the cause of reverse epidemiology, it does not explain the inverse relationship between obesity and mortality in hemodialysis patients. Furthermore, there is a possibility that the interventions causing weight gain in hemodialysis patients result in better survival regardless of the direction of the causal pathway [5].

Other mechanisms

Endotoxin lipoprotein interactions [6,13], survival bias, and time discrepancies are competitive risk factors [13]. Factors affecting the obesity-survival paradox are as follows.

Race

Ricks et al. [14] compared the obesity paradox in three different ethnic groups in his retrospective cohort, including blacks, Hispanics, and non-Hispanic whites. The results suggest that higher BMI is linked with better survival in all the three ethnic groups. However, blacks and Hispanics experienced better survival gains when compared with non-Hispanic whites among the higher BMI categories. The suggested reasons for this finding include a relatively higher muscle mass in blacks at any given BMI compared to other ethnicities; a relatively higher food intake in blacks compared to other races; as well as disparities in health status, income, and education.

Age

Hoogeveen et al. found that younger HD patients (<65 years) with BMI >30kg/m2 have a twofold increase in mortality rate compared with normal weight. This association between mortality and obesity is even more pronounced among patients <50 years of age. [15]

Even a low BMI is associated with high mortality in this age group. The inverse relationship between BMI and all-cause mortality is more evident where the average age of individuals is ≥60 years. Older HD patients have a high mortality rate, and the short-term effects of competitive risk factors like infection, underweight, and MIC syndrome might block the long-term effects of obesity. Hence, obesity is not a risk factor for mortality in these patients.

Gender

A prospective study conducted in Korea [16] showed that male HD patients with BMI above 25.1kg/m^2^ have higher survival than those with BMI less than 25.1kg/m^2^, (p<0.001). Whereas no statistical significance was found in the survival of female patients based on BMI.

Severity of Comorbidities and Intensity of Treatment

Obese and overweight patients may have less severe comorbidities when compared with malnourished patients. Obese patients have demonstrated a better survival rate than normal-weight patients with comparable severity of heart failure after adjusting for age, the severity of illness, and gender. 

Obese and overweight patients are considered to be diagnosed with cardiovascular disease at the earlier stage of their illness due to accentuation of the symptoms of heart failure because of obesity. As a result, they may receive treatment earlier and more intensely than patients with lower BMI [17].

Short-Term Effect

In third-world countries, undernutrition is an important cause of morbidity and mortality, resulting in a shorter life expectancy. Likewise, the short-term survival benefits that exist in obese maintenance hemodialysis patients may outweigh the adverse effects of obesity on cardiovascular disease in the long term. Hence, there is a possibility that obese patients might have a higher survival rate in the short term but not necessarily in long run [9]. This is supported by a long-term study conducted over 12 years by Kaizu et al. [18], which showed no obesity survival paradox, on the other hand, a short-term study conducted over three years by Yen et al. [11], suggested the existence of obesity survival paradox.

Muscle Mass

In a study by Beddhu et al. [19], it was observed that the protective effect of high BMI has been restricted to the patients with normal or high muscle mass (inferred as low body fat). Patients with high BMI with low muscle mass (inferred as high body fat) have been associated with increased all-cause as well as cardiovascular mortality. Whereas, lean individuals (low muscle mass) have an accelerated mortality rate due to poor nutritional status as well as high levels of inflammation. It is important to note that in these studies, muscle mass was reflected by urinary creatinine. This relation between muscle mass and mortality has been explained by different theories, such as uremic toxins are distributed in the muscle mass compartment, high muscle mass is associated with high nonedematous body water, which dilutes circulatory toxins hence lean individuals have higher levels of uremic toxins.

Acetyl-ghrelin

Acetyl-ghrelin is known to improve cardiovascular outcomes by attenuating pressure-overload-induced cardiac hypertrophy, reversing endothelial dysfunction, and improving overall survival. As an appetite-related hormone, high levels of acetyl ghrelin may also contribute to a better nutritional state in hemodialysis patients. A prospective cohort demonstrated that high levels of AG enhance the positive association between obesity and survival in hemodialysis patients [20].

**Table 1:** Association between BMI and mortality (study characteristics table)

Index	Author/year	Study type	Number of patients/number of studies included	BMI Category (kg/m2)	Mortality (%)
1.	Yen et al. 2009 [11]	cohort	959	<18.5	21.6%
				18.5-22.9	13.0%
				23.0-24.9	20.3%
				>25	15.5%
2	Beddhu et al. 2007 [10]	Cohort	1000	<21.8	32.3%
				21.8-25.0	24.8%
				25.1-28.7	23.9%
				>28.7	18.5%
3	Herselman et al.2010 [9]	Systematic review	31 articles		
4	Doshi et al. 2016 [8]	Cohort	123624	18-23	14.9%
				23.1-27.5	11.9%
				27.6-40	10.7%
				>40	1.2%
5	Leavey et al. 2001 [17]	Cohort	9714	<20	26.9%
				20-24.9	20.9%
				25-29.9	17.4%
				>=30	14.1%
6	Beddhu et al. 2003 [19]	Cohort	70028	18.5-24.9	23.3%
				>=25	19.5%
7	Jialin et al. 2012 [5]	Meta-analysis	4 studies	-	-
8	Agarwal et al. 2011 [12]	Cohort	368	<25	13.3%
				25-30	9.2%
				30.1-35	6.25%
				>=35	3.5%
9	Beberashvili et al. 2017 [20]	Prospective Cohort	261	<26.8	24.9%
				>=26.8	16.8%
10	Hoogeveen et al. 2011 [15]	Prospective Cohort	1749	<20	3.7%
				20-24	21.3%
				25-29	12%
				>=30	4.8%
11	Kalantar-Zadeh et al. 2005 [13]	Cohort	54535	<20	4.6%
				20.0-24.99	9.94
				25-29.99	6.18%
				30-39.99	3.5%
				>=40	0.54%

Limitations

The present study has certain limitations, which are mainly attributed to the limitations of the studies that have been included. Our paper is primarily based on observational studies, as randomized controlled trials are not possible with such a study. Most of the studies were conducted retrospectively. We only included papers in the English language. Not all the studies have reported body weight according to the standard WHO/NIH BMI classification system. Even though BMI is commonly used for nutritional assessment surveys as a marker of nutritional status and body size, it is not the best indicator of body composition, as it does not differentiate muscle mass or body water from adiposity. Confounders such as rate, comorbid conditions, acute illnesses, and hospitalizations, were not captured in all the databases.

## Conclusions

Contrary to the general population, this systematic review demonstrates the evidence of an inverse relationship between BMI and all-cause mortality in ESRD patients on HD and we can say that the "Obesity -survival paradox" is real and not just a myth. There are no clear survival mechanisms identified; instead, multiple proposed hypotheses need better understanding and further research is warranted.
